# Relationship between HIV stage and psychomotor speed neurocognitive score at a Kenyan sub-county hospital

**DOI:** 10.4102/phcfm.v8i1.1061

**Published:** 2016-08-31

**Authors:** Rachael N. Kinuthia, Joseph M. Thigiti, Benson N. Gakinya

**Affiliations:** 1 Department of Family Medicine, Moi University, Eldoret, Nairobi; 2 Department of Family Medicine, Kenyatta University College of Health Sciences, Nairobi; 3 Department of Mental Health, School of Medicine, Moi University, Eldoret, Nairobi

## Abstract

**Background:**

Human immunodeficiency virus (HIV) and acquired immunodeficiency syndrome (AIDS) is associated with cognitive impairment which affects psychomotor speed. Psychomotor slowing is a predictor of dementia and death in people living with HIV and AIDS. The purpose of this study was to assess the relationship between HIV disease stage and psychomotor speed neurocognitive score which will add to the body of knowledge required to manage patients with HIV and AIDS.

**Objective:**

To determine the relationship between psychomotor speed neurocognitive score and the HIV disease stage in adults at initiation of care.

**Setting:**

This study was conducted at Kangundo Sub-county hospital comprehensive care centre.

**Methods:**

This was a cross-sectional study. All HIV seropositive patients aged 18 to 50 years recently initiated into care were studied. A pretested questionnaire was used to collect data. The World Health Organization (WHO) stage was used during data collection to classify study participants into asymptomatic and symptomatic groups. The grooved pegboard test was used to obtain psychomotor speed neurocognitive scores. Descriptive statistics were used to summarise data. Mann–Whitney U test, Spearman’s rho and multiple linear regression were employed in the analysis; *p*-value of 0.05 was considered significant.

**Results:**

The WHO stage did not have a significant effect on the psychomotor speed neurocognitive score (*p* ≥ 0.05). The CD4 count had a significant effect on psychomotor speed neurocognitive score (*p* = 0.001).

**Conclusions:**

There was a significant correlation between CD4 counts and psychomotor speed neurocognitive score. Efforts should be made to ensure that the CD4 counts of people living with HIV and AIDS do not continue to fall after initiation into care in order to preserve psychomotor function.

## Introduction

According to Vivithaporn et al., half of HIV-positive patients treated with combination antiretroviral therapy have cognitive impairment. In this era of combination antiretroviral therapy, severe forms of cognitive impairment are rare.^1^ Antinori et al. in previous studies classified HIV-associated neurocognitive disorders into asymptomatic neurocognitive impairment, mild neurocognitive disorder and HIV-associated dementia.^2^

Psychomotor speed is one of the cognitive domains affected by HIV infection. Stern et al. found that abnormal scores in psychomotor speed have been significantly associated with time to develop HIV-associated dementia.^3^ A high viral load in HIV infection has been linked with decline in psychomotor speed by Sacktor et al. Combination antiretroviral therapy has been linked with improved performance in psychomotor speed, memory and executive function.^4,5^ In other studies, Hinkin et al. and Becker et al. have proposed that HIV-positive individuals with cognitive dysfunction are at risk for poor medication adherence-especially those with complex drug regimens.^6,7^

Lawler et al. and Robertson et al. pointed out that 67% of people infected with HIV live in sub-Saharan Africa. Cognitive impairment in HIV is less commonly described compared to Central nervous system (CNS) opportunistic infections.^8,9^ Clifford et al. in Ethiopia found minor evidence of HIV-associated neurocognitive deficits which varies from findings of studies done in South Africa by Joska et al. which found that HIV-positive patients performed significantly worse on tests of cognition.^10,11^ Sacktor et al. in Uganda demonstrated that different HIV subtypes may differ in their capacity to cause cognitive impairment.^12^

According to previous studies carried out by Kwasa et al. and Zaheer in Kenya, there are limited data on HIV-associated cognitive impairment due to a limited number of neurologists and limitations in diagnostic capabilities.^13,14^

HIV and AIDS is a leading cause of disability. Almost 40 million people were living with HIV at the end of 2012 all over the world.^4^ Sub-Saharan Africa remains the most severely affected region with almost 1 in 20 adults living with HIV and accounting for two-thirds of people living with HIV globally.^15^ In Kenya, according to the Kenya AIDS indicator survey 2012, HIV prevalence was 5.6%.^16^

Neurocognitive impairment in HIV infection has emerged as a very important issue over the years.^8^ Psychomotor speed is one of the cognitive domains significantly affected by cognitive impairment in People Living With HIV and AIDS (PLWHA) and can be used to predict cognitive impairment, dementia and death.^17^ Abnormal scores in psychomotor speed have been associated with time to develop severe cognitive impairment in HIV.^3^ PLWHA who have deficits in psychomotor function are at risk of poor medication adherence especially when complex drug regimens are prescribed.^6,7^ In Kenya, diagnosis of HIV-associated cognitive impairment is a challenge in primary care settings, and there are limited data on cognitive impairment in HIV.^14^ Psychomotor slowing is an early symptom of HIV-associated neurocognitive impairment which affects adherence to medications and increases the chances of high-risk behaviour that could lead to new infections if unrecognised. ^8,18,19^ HIV-associated neurocognitive impairment can be treated by the use of combination antiretroviral therapy which can halt progression or reverse symptoms. ^18,20^ As psychomotor slowing is a predictor of progression to AIDS and death, it is important to understand the relationship between disease stage and psychomotor speed neurocognitive score at baseline so as to guide the clinician in patient management. No study has looked at the relationship between psychomotor speed neurocognitive score and the World Health Organization (WHO) HIV disease stage in Machakos County.

Determining the relationship between the psychomotor speed and HIV disease stage will help add to the body of knowledge required in the management of people living with HIV and AIDS in order to improve quality of life and health outcomes.

In this study, the WHO HIV and AIDS staging system was used to classify participants into four stages (stage 1 to 4). Participants were further classified into asymptomatic and symptomatic disease stages depending on their WHO stage. Those who fell in stage 1 and 2 were classified as asymptomatic while those in stage 3 and 4 are referred to as symptomatic.

### Aim of the study

To determine the relationship between the HIV and AIDS stage, CD4 count and the psychomotor speed neurocognitive score at initiation of care at Kangundo Sub-county hospital comprehensive Care centre.

### Specific objectives

- Determine the patient’s WHO disease stage and the baseline CD4 count at initiation into care.
- Determine the psychomotor speed neurocognitive score of HIV seropositive patients at the time of initiation into care.
- Determine association between HIV and AIDS WHO stage and psychomotor speed neurocognitive score.
- Determine association between baseline CD4 count and the psychomotor speed neurocognitive score.

## Research methods and design

### Study design

This was a cross-sectional study.

### Setting

The study was conducted at Kangundo Sub-county hospital; this hospital has a bed capacity of 174. Kangundo Sub-county hospital is a level-4 hospital in Machakos County about 70 km to the east of the capital city of Kenya, Nairobi. The hospital has a catchment population of 229,485 people.

### Study population and sampling strategy

All adult HIV-positive patients recently initiated into care at Kangundo Sub-county hospital who met the inclusion criteria were eligible. This hospital’s comprehensive care centre (CCC) attends to an average of 60 people daily. The CCC has 3,269 registered PLWHA on care and 1,970 on HAART (39%). The study population comprised 18- to 50-year-old adult HIV seropositive patients recently initiated into care, and those who were already on HAART were excluded as the aim of this study was to assess the baseline psychomotor speed at the start of treatment. Those who had history of past or current substance abuse, chronic psychiatric illnesses and history of head injury were excluded as this may act as confounding factors (Box 1).

BOX 1 World Health Organization HIV and AIDS disease stage.

**Table:** 

Asymptomatic stage or early stage		Symptomatic stage or late stage	
WHO stage 1	WHO stage 2	WHO stage 3	WHO stage 4
Asymptomatic Persistent generalised lymphadenopathy (PGL)	Moderate unexplained weight loss (less than 10% of body weight) Recurrent respiratory tract infections Herpes zoster Angular cheilitis Recurrent oral ulcerations Popular pruritic eruptions Seborrheic dermatitis Fungal nail infections of fingers	Severe weight loss more than 10% of body weight Unexplained chronic diarrhoea for more than one month Unexplained fever longer than one month Oral candidiasis Oral hairy leukoplakia Pulmonary tuberculosis diagnosed for last two years Severe presumed bacterial infections (e.g. pneumonia, empyema, pyomyositis, joint infection, meningitis and bacteraemia) Acute necrotising ulcerative stomatitis, gingivitis or periodontitis. Unexplained anaemia of less than 8 g/dl, and or neutropenia of less than 500/mm^3^ and or thrombocytopenia (<50 000/mm^3^) for more than a month.	HIV wasting syndrome Pneumocystis pneumonia Recurrent severe or radiological pneumonia Chronic herpes simplex infection Oesophageal candidiasis Extra pulmonary TB Kaposi’s sarcoma Central nervous system (CNS) toxoplasmosis HIV encephalopathy Extra pulmonary cryptococcosis including meningitis Disseminated non-tuberculous mycobacteria infection Progressive multifocal leucoencephalopathy Candida of trachea, bronchi or lungs Cryptosporidiosis Isosporiasis Visceral herpes simplex infection Cytomegalovirus infection Any disseminated mycosis (e.g. histoplasmosis, coccidiomycosis and penicillosis) Recurrent non-typhoidal salmonella septicaemia Lymphoma (cerebral or B cell non-Hodgkin’s) Invasive cervical carcinoma Visceral leishmaniasis

*Source*: Information provided in this box was obtained from the revised who clinical staging of HIV and AIDS for adults and adolescents

Information provided in this table was obtained from the revised who clinical staging of HIV and AIDS for adults and adolescents.

To measure and compare the proportion of patient with psychomotor slowing in both the late (symptomatic) and early (asymptomatic) HIV and AIDS, the sample size estimation formula was

$$\frac{N=\left(Z\alpha /2+Z1-\beta \right)2\left(\text{P}1\left(1-\text{P}1\right)+\text{P}2\left(1-\text{P}2\right)\right)}{\left(\text{P}1-\text{P}2\right)2}$$ [Eqn 1]

where:

P1 = Proportion of PLWHA expected to have psychomotor slowing in early (asymptomatic) HIV.

P2 = Proportion of PLWHA expected to have psychomotor slowing in late (symptomatic) HIV.

*Z*α = the standard normal deviate for α.

*Z*β = the standard normal deviate for β.

*N* = Total number of subjects (sample size)

Since choice reaction time has been shown to correlate with the grooved pegboard test (0.47–0.62), prevalence obtained from a Nigerian study that utilised choice reaction time to assess psychomotor speed was used.^21, 22^

In this study, using values obtained from the Nigerian study, P1 = 0.385, P2 = 0.827, *Z*α = 0.05, *Z*β = 0.9 and *N* = 72 the total sample consisted of 36 patients in early HIV and 36 patients in late HIV (total of 72).

Consecutive sampling was used to recruit participants in both asymptomatic (early HIV disease) and symptomatic (late HIV disease); this is because the sampling frame was unknown. Recruitment into the two groups run concurrently as the new patients came into the comprehensive centre and were assessed and recruited into either asymptomatic or the symptomatic group. The WHO disease stage was not previously known and was assigned by the principal investigator and her research assistant following history taking and physical examination of each of the participants after which laboratory workup was done to obtain among other investigations the CD4 counts for each of the participants.

### Data collection

The data was collected by the principal investigator and a trained research assistant. A pretested structured questionnaire was used to collect biodata and record the patient WHO stage. The grooved pegboard test was used to determine psychomotor neurocognitive scores. Blood samples were obtained from the patient and taken to the hospital laboratory for the measurement of CD4 counts and these were recorded on corresponding questionnaire. Psychomotor speed neurocognitive scores were obtained using the grooved pegboard test. The grooved pegboard test was chosen to test for psychomotor speed in this study because it does not require the participant to know how to write and therefore is easier to administer compared to other tests. The test is sensitive for general slowing with the non-dominant hand being more sensitive in detecting psychomotor slowing compared to the dominant hand. The grooved pegboard test has two trials: the dominant and the non-dominant hand trials. The participant was asked to place 25 keyed pegs into an array of 25 slotted holes as fast as possible; both hands were tested separately. The dominant hand was assumed to be the hand used by the participant to write. Dominant hand trial was offered first followed by the non-dominant hand trial. The score was recorded separately for both hands and reflected number of seconds taken to complete the task.^23^ Using the accompanying grooved pegboard test manual, *Z*-scores were calculated from the scores obtained in the test using the formulae:

*Z*-Score = Total Time minus the Age Mean divided by the Standard Deviation.

Negative *Z*-scores on this task (the participant performing in less time than the age mean) demonstrate performance in the average to superior range, whereas positive *Z*-scores on this task (the participant performing in more time than the age mean) demonstrate performance in the average to well-below-average range. The grooved pegboard test manual provides normative data which are age- and sex-specific. Participant with *Z*-scores of more than 1.5 standard deviations were classified as having abnormal scores. ^23,24^

### Data analysis

Questionnaires were checked for completeness and errors. Information from completed questionnaires was entered in a database designed in Epidata, a data entry software, version 3.1 and later exported to SPSS version 17 for analysis.

Descriptive statistics (percentages, means, frequencies, medians, interquartile range) were used to summarise data.

Non-parametric Mann–Whitney U test was used to compare the medians because the data was skewed.

Spearman’s rho was used to assess for correlation between age and psychomotor speed neurocognitive scores.

Multiple linear regression analysis was used to assess for linear relationship between WHO stages, CD4 count, gender, age and psychomotor speed neurocognitive score. *p* < 0.05 was considered significant.

### Ethical considerations

This research was approved by Moi University institutional research and ethics committee (Reference number IREC/2012/168, approval number 000880). Informed consent was obtained from all the participants.

Participants’ information was confidential and was not used for any other purpose other than the study. All interviews were conducted in a secluded room with one individual subject at a time, and the filled questionnaires were kept in a safe custody by the principal investigator in order to ensure that confidentiality was maintained throughout the study.

## Results

Seventy-two HIV seropositive patients participated in the study. Majority of the participants were female, most had attained secondary or primary education. The mean age was 35.5 (±7.6) and 36.0 (±8.3) in the asymptomatic and symptomatic groups, respectively as shown in Table 1.

**TABLE 1:** Social demographic characteristics of the participants.

Variable	Characteristic	Asymptomatic	Symptomatic	*p*
Gender	Male	7 (28%)	18 (72%)	0.013
	Female	29 (61.7%)	18 (38.3%)	
Level of education	None	0	1 (100%)	0.134
	Primary	17 (63%)	10 (37%)	
	Secondary	18 (47.4%)	20 (52.6%)	
	Tertiary	1 (16.7)	5 (83.3%)	
Employment	Yes	17 (45.9 %)	20 (54.1%)	0.84
	No	15 (51.7%)	14 (48.3%)	
Age mean (s.d.)		35.5 (±7.6)	36.0 (±8.3)	0.680

*Source*: The table information was compiled from analysis of data obtained in the study using above tests. *χ*^2^ for gender, level of education and employment, *t*-test for age. s.d., standard deviation

**FIGURE 1:**
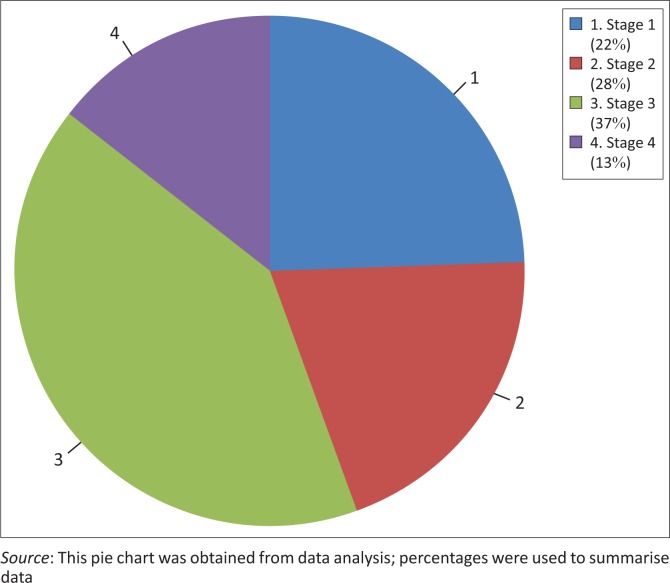
WHO stage (*n* = 72).

Most of the participants in the study were in WHO stage 3 as shown in Figure 1. The median CD4 count was 434 (216.5, 561) in the asymptomatic group. In the symptomatic group, the median CD4 count was 76 (27.25, 296). Majority of the patients in the asymptomatic (88.8%) and symptomatic group (94.4%) reported right hand as the dominant hand.

### HIV by disease stage score (speed)

There was a significant difference in psychomotor speed neurocognitive score in the dominant hand between the symptomatic and asymptomatic groups (*p* = 0.003) as shown in Table 2. There was no significant difference in psychomotor speed neurocognitive score between the symptomatic and asymptomatic groups in the non-dominant hand as shown in Table 2 (*p* = 0.081).

**TABLE 2:** Difference in median psychomotor speed neurocognitive score.

Variable	Dominant hand Median psychomotor speed neurocognitive score (IQR)	Non-dominant hand Median psychomotor speed neurocognitive score (IQR)
Asymptomatic (*n* = 36)	80.5 (68.25, 92.75)	87.5 (79,98.5)
Symptomatic (*n* = 36)	100.5 (79,121.5)	98 (82, 137.5)
*p*	0.003	0.081

*Source*: Authors’ own work. This table was obtained from data analysis; Mann–Whitney U test was used to compare the medians because the data were skewed

### Association between CD4 and psychomotor speed neurocognitive score (speed)

After classifying the CD4 count into below 200 cells/mm^3^ and above 200 cells/mm^3^, there was a significant difference in psychomotor speed score between those with CD4 counts less than 200 cells/mm^3^ and those with more than 200 cells/mm^3^ psychomotor speed as shown in Table 3.

**TABLE 3:** Difference in median psychomotor speed neurocognitive score by patients CD4 count.

Indicator	Speed	
	Dominant hand Median psychomotor speed neurocognitive score (IQR)	Non-dominant hand Median psychomotor speed neurocognitive score (IQR)
CD4		
< 200, *n* = 29	103 (79, 124)	109 (85.5, 138)
≥ 200, *n* = 43	81 (70, 102)	85 (79,96)
*p*	0.006	0.04

*Source*: Authors’ own work. This table was obtained from data analysis; Mann–Whitney U test was used to compare the medians because the data were skewed

As indicated in Table 4, there was no significant difference in psychomotor speed neurocognitive score by gender in both the asymptomatic and symptomatic groups (*p* > 0.05).

**TABLE 4:** Relationship between gender and psychomotor speed neurocognitive score.

Disease category	Median (IQR)		*p*
	Male	Female	
**Asymptomatic**			
Dominant hand	83 (70, 86)	80 (68, 97.5)	0.845
Non-dominant hand	85 (79, 119)	88 (79, 98.0)	0.969
**Symptomatic**			
Dominant hand	119.5 (83.50, 148.5)	94 (72, 106.25)	0.055
Non-dominant hand	120.5 (85.75, 152.0)	86 (78, 119.50)	0.059

*Source*: Authors’ own work. This table was obtained from data analysis; Mann–Whitney U test was used to compare the medians because the data were skewed

A significant weak positive correlation was found between age and psychomotor speed neurocognitive score only in the non-dominant hand in the asymptomatic group as shown in Table 5.

**TABLE 5:** Correlation between age and psychomotor speed neurocognitive score.

Variables	Dominant hand	Non-dominant hand
Asymptomatic	rho = 0.199	rho = 0.360
	*p* = 0.244	*p* = 0.031
Symptomatic	rho = 0.277	rho = 0.161
	*p* = 0.102	*p* = 0.348

*Source*: Authors’ own work. This information was obtained from data analysis using Spearman’s rho to assess for correlation between age and psychomotor speed neurocognitive test in dominant and non-dominant hands. rho, Spearman’s rho

The level of education did not significantly influence psychomotor speed neurocognitive score in both dominant (*p* = 0.42) and non-dominant (*p* = 0.252) hands, as shown in Table 6.

**TABLE 6:** Correlation between education and psychomotor speed neurocognitive score.

Level of education	Dominant hand Median psychomotor speed score (IQR)	Non-dominant hand Median psychomotor speed score (IQR)
None/primary	92 (79,127)	96 (85,134)
Secondary/tertiary	92.5 (67.75, 139)	111 (74.5, 184.25)
*p*	0.420	0.252

*Source*: Authors’ own work. This table was obtained from data analysis; Mann–Whitney U test was used to compare the medians because the data were skewed

It appears multicollinearity is not a concern because the Variance Inflation Factor (VIF) scores are less than 3.

The slope of gender is 0.159. This means that, on average, predicted psychomotor scores for males are 0.159 points higher than for females, after controlling for WHO stage, as indicated in Table 7.

**TABLE 7:** Multiple linear regression (dominant hand).

Model	Standardised coefficients Beta	T	Sig.	Collinearity statistics	
				Tolerance	VIF
Gender (male versus female)^a^	0.270	2.181	0.033	0.783	1.278
WHO1^b^	-0.275	-1.648	0.104	0.431	2.319
WHO2	-0.145	-0.772	0.443	0.342	2.926
WHO3	0.055	0.306	0.761	0.373	2.681

*Source*: Authors’ own work. Multiple linear regression was used to assess for linear relationship between gender, WHO stage and psychomotor speed neurocognitive score

a. Dependent variable: Psychomotor score (Dominant hand),

b. WHO stage 4 is the reference for WHO stage; female gender is the reference for gender

It appears multicollinearity is not a concern because the VIF scores are less than 3. The slope of CD4 is -0.072. This means that for every one unit increase in CD4, predicted psychomotor score decreases by 0.072 units, after controlling for age and gender. This is illustrated in Table 8.

**TABLE 8:** Multiple linear regression (non-dominant hand).

Model	Unstandardised coefficients		Standardised coefficients Beta	T	Sig.	Collinearity statistics	
	B	Standard error				Tolerance	VIF
(Constant)	4.722	0.200	-	23.577	0.000	-	-
Age	0.006	0.005	0.166	1.434	0.156	0.843	1.187
Gender	0.039	0.078	0.060	0.502	0.617	0.781	1.281
CD4	-0.072	0.021	-0.396	-3.513	0.001	0.885	1.129

*Source*: Authors’ own work. Chi-square test was used in data analysis to compare percentages of abnormal as well as normal scores across the WHO disease stage. Dependent variable: Psychomotor speed (non-dominant hand)

After conversion of the raw scores into *Z*-scores and classifying participants into those with *Z*-scores of 1.5 s.d. above the mean and those whose *Z*-scores fell 1.5 s.d. below the mean, there was no significant difference between percentages of those with abnormal score in the asymptomatic and symptomatic groups as illustrated in Table 9 in both dominant (*p* = 0.454) and non-dominant (*p* = 0.637) hands.

**TABLE 9:** Percentage of normal versus abnormal scores by disease category.

Variable	Dominant hand			Non-dominant hand		
	Normal (%)		Abnormal (%)	Normal (%)		Abnormal (%)
WHO stage 1 and 2 (asymptomatic)	14 (38.9)		22 (61.1)	18 (50.0)		18 (50.0)
WHO stage 3 and 4 (symptomatic)	10 (27.8)		26 (72.2)	15 (41.7)		21 (58.3)
*p*		0.454			0.637	

*Source*: Authors’ own work. Chi-square test was used in data analysis to compare percentages of abnormal as well as normal scores across the CD4 categories

Participants were also classified into those with normal and abnormal scores depending on CD4 count category; there was no significant difference between those with abnormal scores in asymptomatic and symptomatic groups in both dominant (*p* = 0.077) and non-dominant (*p* = 0.054) hands as seen in Table 10.

**TABLE 10:** Percentage of normal versus abnormal scores by CD4 count category.

CD4 category	Dominant hand			Non-dominant hand		
	Normal (%)		Abnormal (%)	Normal (%)		Abnormal (%)
CD4 < 200	6.0 (20.7)		23.0 (79.3)	9 (31)		20 (69)
CD4 > 200	18.0 (41.9)		25 (58.1)	24 (55.8)		19 (44.2)
*p*		0.077			0.054	

*Source*: Authors’ own work. Chi-square test was used in data analysis to compare percentages of abnormal as well as normal scores across the CD4 categories

## Discussion

In this study, the WHO stage did not have a significant effect on the psychomotor speed (*p* > 0.05). The CD4 count had a significant effect on psychomotor speed (*p* = 0.001). The majority of participants in this study were women; this could be attributed to health-seeking behaviour as previous research has shown that there are important differences between women and men in underlying mechanisms of HIV and AIDS infection as well as the social and economic consequences which arise from differences in biology, sexual behaviour and socially constructed gender differences.^25^ Responsibilities, gender roles, access to resources and decision-making power may result in women being more vulnerable to HIV compared to men.^26,27,28^

The mean age was 35.5 (±7.6) and 36.0 (±8.3) in the asymptomatic and symptomatic groups, respectively. This is consistent with findings from studies which have shown that HIV and AIDS affect people in their prime years.^29,30^ Studies done in South Africa revealed that most deaths due to HIV and AIDS occurred in people between 29 and 40 years of age.^29^

Most of the participants had attained a primary or secondary school education. Past research has demonstrated that education alone may not prevent people from acquiring HIV infection; other factors like income and religion interact with education to influence HIV infection rates.^31^

With regard to employment status, about half of the participants in symptomatic and asymptomatic disease stages were in some form of employment. This may be explained by the fact that this study took place in a rural area and bearing in mind that most of the participants were women, they were likely to be housewives or farmers or self-employed. PLWHA may also find it difficult to continue full-time employment due to challenges which include job discrimination and ongoing medical needs which necessitate a change of schedules.^32^ This may also explain why half of the respondents were not in full-time employment.

During this study, participants were able to understand instructions on the grooved pegboard test and complete the test.

### Association between CD4 count and psychomotor speed neurocognitive score

In this study, there was a significant association between CD4 count and the psychomotor speed neurocognitive score after controlling for effects of age and gender. The psychomotor speed neurocognitive score is the time taken to complete test in seconds. The psychomotor speed score decreased by 0.072 for every one unit increase in CD4 count such that those with higher CD4 counts took lesser time to complete the test as compared with those whose CD4 counts are lower. This differs from a previous study which compared test outcomes for participants with CD4 counts and found no significant difference. This finding could be attributed to the fact that the study recruited HIV-1 seroconverters who may have had higher levels of CD4 counts compared to this study and therefore did not exhibit clinically significant impairment in cognitive performance. About 5.1% of the participants were on antiretroviral therapy which could also have had a protective effect and therefore affected the results obtained.^33^

### Relationship between HIV disease stage and psychomotor speed neurocognitive score

Although initially there was a significant association between the HIV disease stage and psychomotor speed neurocognitive score in the dominant hand, this was not found to be the case in the non-dominant hand. This could be due to the practice effect so that after being offered the dominant hand trial, the participants became more confident and were able to perform the non-dominant hand trial within a shorter time compared with the time used to complete the dominant hand trial. This is consistent with findings from a previous study which showed that lack of prior exposure to neuropsychological tests may affect performance.^33^

After multiple regression analysis, there was no significant difference in psychomotor speed neurocognitive score between the asymptomatic and symptomatic HIV disease stages. This differs from the findings of Kanmogne et al. who found that advancing disease stage significantly affected performance in a study done in Cameroon where the grooved pegboard test was also used to assess motor function. Study participants with AIDS were found to perform poorly compared to those in the non-AIDS group.^34^ The findings of this study also differ from those of Heaton et al. who found that grooved pegboard test completion time was related to stages of HIV infection.^35^ The differences in finding could be attributed to ethnic or cultural differences across groups which have been noted in previous studies to affect performance in neuropsychological tests. The HIV and AIDS disease classification used by Kanmogne et al. was the centre for disease control staging which also takes into account the CD4 count; those participants with CD4 counts of less than 200 are in the advanced disease stage. The effect of advancing disease observed in their study could have been due to falling CD4 count which was found to affect psychomotor speed score in this study. In our study, the WHO disease staging was used and did not take into account the CD4 count.^34^

### Demographic effects

Other studies have noted that demographic effects, age, education level and gender may affect performance in neuropsychological tests.^34^ In this study, gender was found to affect performance in psychomotor speed neurocognitive scores as demonstrated in the multiple linear regression analysis. This differs from finding in another study which did not find any significant effect of gender on test performance. The grooved pegboard was also used as part of the neuropsychological test battery to test for fine motor skills and dexterity.^34^

Older age has been associated with poor performance in previous studies.^33,34^ Findings in this study show that age did not have a significant effect on psychomotor speed neurocognitive scores.

Lower education levels have been associated with poor neuropsychological test performance in previous studies; in this study, education did not significantly affect test performance.^33^

### Percentage of participants with abnormal scores depending on disease stage and CD4 count

In this study, normative scores from the general US population were used to convert raw scores obtained from the grooved pegboard test into standardised scores (*z*-scores) because there is currently no published normative data from Kenya or East Africa.^14^ There was no significant difference observed between percentages of those with abnormal scores between those in asymptomatic or symptomatic groups (*p* > 0.05), there was also no significant difference in percentages of abnormal scores between those with CD4 counts of less than 200 and more than 200 cells per cubic millimetre. This may be explained by the findings from previous research which showed that normal individuals were classified as abnormal when normative data from the United States was used to convert raw scores into *Z*-scores which could have affected the percentages of abnormal scores in both asymptomatic, symptomatic participants groups and both CD4 cell categories.^14^ As CD4 count correlates with psychomotor speed neurocognitive score and falling CD4 adversely affects psychomotor speed scores, care should be taken to ensure that CD4 counts do not continue falling after initiation into care in order to preserve psychomotor function in people living with HIV and AIDS. There is need for development of culturally appropriate normative scores for the grooved pegboard test before further use in research.

## Conclusions

There was no significant correlation between WHO stage and psychomotor speed neurocognitive score. There was a significant correlation between CD4 count and psychomotor speed neurocognitive score.

## References

[1] Vivithaporn P, Heo G, Gamble J, et al Neurological burden in treated HIV and AIDS predicts survival, a population based study. Neurology. 2010;75(13):1150–1158. http://dx.doi.org/10.1212/WNL.0b013e3181f4d5bb 20739646 10.1212/WNL.0b013e3181f4d5bb PMC3013488

[2] Antinori A, Arendt G, Becker JT, et al Updated research nosology for HIV-associated neurocognitive disorders. Neurology. 2007;69(18):1789–1799. http://dx.doi.org/10.1212/01.WNL.0000287431.88658.8b 17914061 10.1212/01.WNL.0000287431.88658.8b PMC4472366

[3] Stern Y, McDermott MP, Albert S, et al Factors associated with incident human immunodeficiency dementia. Arch Neurol. 2001;58(3):473–479. http://dx.doi.org/10.1001/archneur.58.3.473 11255452 10.1001/archneur.58.3.473

[4] Sacktor N, Skolasky RL, Tarwater PM, et al Response to system HIV viral load suppression correlates with psychomotor speed performance. Neurology. 2003;61(4):567–569. http://dx.doi.org/10.1212/01.WNL.0000076477.71313.6E 12939443 10.1212/01.wnl.0000076477.71313.6e

[5] Sacktor N, Nakasujja, Skolasky RN, et al Antiretroviral therapy improves cognitive impairment in HIV positive individuals in Sub-Saharan Africa. Neurology. 2006;67(2):311–314. http://dx.doi.org/10.1212/01.wnl.0000225183.74521.72 16864825 10.1212/01.wnl.0000225183.74521.72

[6] Hinkin CH, Castellon SA, Durvasula RS, et al Medication adherence among HIV positive adults. Effects of cognitive dysfunction and regimen complexity. Neurology. 2002;59(12):1944–1950. http://dx.doi.org/10.1212/01.WNL.0000038347.48137.67 12499488 10.1212/01.wnl.0000038347.48137.67 PMC2871670

[7] Becker BW, Thames AD, Woo E, Castellon SA, Hinkin CH Longitudinal change in cognitive and medication adherence in HIV infected adults. AIDS Behav. 2011;15(8):1888–1894. http://dx.doi.org/10.1007/s10461-011-9924-z 21437726 10.1007/s10461-011-9924-z PMC3616486

[8] Lawler K, Jeremiah K, Mosepele M, Ratcliffe SJ, Cherry C, Seloilwe E, Steenhoff AP Neurobehavioral effects in HIV-positive individuals receiving highly active antiretroviral therapy (HAART) in Gaborone, Botswana. PLoS One. 2011 Feb 18;6(2):e17233. 21365002 10.1371/journal.pone.0017233 PMC3041805

[9] Robertson K, Liner J, Hakim J, et al NeuroAIDS in Africa. J Neurovirol. 2010;16(3):189–202. http://dx.doi.org/10.3109/13550284.2010.489597 20500018 10.3109/13550284.2010.489597 PMC3549534

[10] Clifford DB, Mitike MT, Mekonnen Y, et al Neurological evaluation of untreated human immunodeficiency virus infected adults in Ethiopia. J Neurovirol. 2007;13:67–72. http://dx.doi.org/10.1080/13550280601169837 17454450 10.1080/13550280601169837

[11] Joska JA, Westgarth–Taylor J, Myer L, et al Characterisation of HIV-associated neurocognitive disorders among individuals starting antiretroviral therapy in South Africa. AIDS Behav. 2011;15(6):1197–1203. http://dx.doi.org/10.1007/s10461-010-9744-6 20614176 10.1007/s10461-010-9744-6

[12] Sacktor N, Nakasujja N, Robertson K, Clifford DB HIV-associated cognitive impairment in Sub-Saharan Africa – the potential effect of clade diversity. Nat Clin Pract Neurol. 2007;3(8) 436–443. http://dx.doi.org/10.1038/ncpneuro0559 17671521 10.1038/ncpneuro0559

[13] Zaheer MAB Cognitive dysfunction among HIV-positive patients attending comprehensive care clinic at Kenyatta National Hospital [unpublished doctoral thesis] University of Nairobi; 2011.

[14] Kwasa J, Cettomai D, Lwanya E, et al Lessons learnt developing a diagnostic tool for HIV-associated dementia feasible to implement in resource limited settings: Pilot testing in Kenya. PLoS One. 2012, 7(3). http://dx.doi.org/10.1371/journal.pone.0032898 10.1371/journal.pone.0032898 PMC3296754 22412945

[15] Holguin A, Banda M, Willen EJ, Malama C, Chiyenu KO, Mudenda VC, Wood C HIV-1 effects on neuropsychological performance in a resource-limited country, Zambia. AIDS and Behavior. 2011 Nov 1;15(8):1895–901. 21744118 10.1007/s10461-011-9988-9 PMC3314062

[16] Kenya AI Indicator survey 2012. Preliminary Report. [http://nascop.or.ke/library/3d/PreliminaryReportforKenyaAIDSindicatorsurvey2012.pdf]. 2014

[17] Sacktor NC, Bacellar H, Hoover DR, et al Psychomotor slowing is a predictor of dementia, AIDS and death. J Neurovirol. 1996;2(6):404–410. http://dx.doi.org/10.3109/13550289609146906 8972422 10.3109/13550289609146906

[18] Sacktor N, Skolasky RL, Cox C, et al Longitudinal psychomotor speed-performance in Human immunodeficiency virus seropositive individual: Impact of age and serostatus. J Neurovirol. 2010;16(5):335–341. http://dx.doi.org/10.3109/13550284.2010.504249 20726699 10.3109/13550284.2010.504249 PMC3068912

[19] Baldewicz TT, Leserman J, Silva SG, et al Changes in neuropsychological functioning with progression of HIV-1 infection: Results of an 8 year longitudinal investigation. AIDS Behav. 2008;8(3):345–355. http://dx.doi.org/10.1023/B:AIBE.0000044081.42034.54 15475681 10.1023/B:AIBE.0000044081.42034.54

[20] Von Giesen HJ, Köller H, Theisen A, Arendt G Therapeutic effects of nonnucleoside reverse transcriptase inhibitors on the central nervous system in HIV-1-infected patients. J Acquir Immune Defic Syndr. 2002;29(4):363–367. http://dx.doi.org/10.1097/00126334-200204010-00006 11917240 10.1097/00126334-200204010-00006

[21] Cysique LA, Manif P, Darby D, Brew BJ The assessment of cognitive function in advanced HIV-1 infection and AIDS dementia complex using computerised cognitive test battery. Arch Clin Neuropsychol. 2006;21(2):185–194. http://dx.doi.org/10.1016/j.acn.2005.07.011 16343841 10.1016/j.acn.2005.07.011

[22] Ogunrin AO, Odiase FE, Ogunniyi A Reaction time in patients with HIV and AIDS and correlation with CD4 count, a case control study. Tran R Trop Med Hyg. 2007;101(5):517–522. http://dx.doi.org/10.1016/j.trstmh.2006.10.002 10.1016/j.trstmh.2006.10.002 17254620

[23] Davis HF, Skolasky RL Jr, Selnes OA, Burgess DM, McArthur JC Assessing HIV-associated: Modified HIV dementia scale versus the grooved pegboard. AIDS Read. 2002;12(1):29–32. 11862658

[24] Instrument L Instruction manual for the 32025 Grooved pegboard test. Lafayette Instrument, Lafayette, USA. 1989.

[25] World Health Organization The world health report 2003: shaping the future. World Health Organization; 2003 World Health Organization. Gender and HIV and AIDS. WHO; 2003.

[26] Mills EJ, Singh S, Nelson BD, Nachega JB The impact of conflict on HIV/AIDS in sub-Saharan Africa. Int J STD AIDS. 2006 Nov 1;17(11):713-7. http://dx.doi.org/10.1258/095646206778691077 17062170 10.1258/095646206778691077

[27] De Vogli R, Birbeck GL Potential impact of adjustment policies on vulnerability of women and children to HIV and AIDS in Sub-Saharan Africa. J Health Popul Nutr. 2005;23(2):105–120. 16117362

[28] Ashford L How HIV and AIDS affects population. World. 2006;38–600.

[29] Fouad DM Role of elderly people in the era of HIV/AIDS in Africa. Global Action on Aging, 2005; p. 1–18.

[30] Njororai F, Njororai WWS Older adult caregivers to people with HIV/AIDS in Sub-Saharan Africa: A review of literature and policy implications for change. Int J Health Promo Educ. 2013;51(5):248–266. http://dx.doi.org/10.1080/14635240.2012.703389

[31] Brent RJ Does female education prevent the spread of HIV-AIDS in Sub-Saharan Africa. Appl Econ. 2006;38(5):491–503. http://dx.doi.org/10.1080/00036840500392045.

[32] Brooks RA, Klosinski LE Assisting persons living with HIV/AIDS to return to work: Programmatic steps for AIDS service organizations. AIDS Educ Prev. 1999 Jun 1;11(3):212. 10407455

[33] Vo QT, Cox C, Li X, et al Neuropsychological test performance before and after HIV-1 seroconversion, the multi AIDS cohort study. J Neurovirol. 2013;19(1):24–31. http://dx.doi.org/10.1007/s13365-012-0136-8 23229349 10.1007/s13365-012-0136-8 PMC3568242

[34] Kanmogne GD, Kuate CT, Jacobson LP, et al HIV-associated disorders in Sub-Saharan Africa; a pilot study in Cameroon. BMC Neurol. 2010;10(1):60 http://dx.doi.org/10.1186/1471-2377-10-60 20626870 10.1186/1471-2377-10-60 PMC2912842

[35] Heaton RK, Grant I, Butters N, et al The HNRC 500 – neuropsychology of HIV infection at different stages. J Int Neuropsychol Soc. 1995;1(3):231–251. http://dx.doi.org/10.1017/S1355617700000230 9375218 10.1017/s1355617700000230

